# Cesarean delivery in a patient with inclusion body myositis: is general anesthesia safe? A case report

**DOI:** 10.3389/fmed.2026.1799325

**Published:** 2026-05-25

**Authors:** Yanan Pu, Yang Su, Liya Li, Mei Han, Jiayuan Niu, Hui Ma

**Affiliations:** Department of Anesthesiology, The Second Affiliated Hospital of Dalian Medical University, Dalian, China

**Keywords:** anesthetic management, case report, cesarean delivery, general anesthesia, inclusion body myositis

## Abstract

**Introduction:**

Inclusion body myositis (IBM) is a rare inflammatory myopathy characterized by progressive limb muscle weakness, dysphagia, and respiratory impairment. In this report, we review the case of a pregnant patient with IBM who underwent cesarean section safely under general anesthesia. Perioperative anesthetic management for these patients has been rarely described.

**Case presentation:**

We describe a 43-year-old parturient (G2P1) who was diagnosed with IBM 7 years prior to this pregnancy, with confirmed involvement of the flexor digitorum profundus and quadriceps muscles. She had mild dysphagia but no respiratory muscle involvement. Due to gestational diabetes mellitus and fetal macrosomia, she required a cesarean section. General anesthesia was administered with aspiration precautions and prophylaxis against malignant hyperthermia (MH). General anesthesia, combined with a reduced dose of rocuronium and reversal with sugammadex, facilitated uneventful extubation within 30 min postoperatively. The newborn was assigned a 10/10 Apgar score at 1 and 5 min after delivery, and the patient was discharged on postoperative day 4 without complications.

**Conclusion:**

This case demonstrates that, with precautionary preparations targeting potential complications—including aspiration risk, MH, exaggerated sensitivity to neuromuscular blocking agents (NMBAs), and postoperative pulmonary complications—general anesthesia may be a safe and feasible option for cesarean delivery in IBM patients.

## Introduction

Inclusion body myositis (IBM) is a rare, idiopathic, inflammatory myopathy with clinically unique pathological features, including rimmed vacuoles with granular material in muscle, atrophic fibers, and eosinophilic cytoplasmic inclusions (1). Clinically, IBM is characterized by slowly progressive asymmetric weakness of the proximal lower extremity (quadriceps) or distal upper extremity (wrist and flexor digitorum profundus) with no associated sensory deficits (2, 3). Dysphagia is also a common complication in patients with IBM, which increases their risk of aspiration (4). More critically, respiratory muscle involvement in IBM frequently progresses to respiratory insufficiency: specifically, impaired inspiratory muscle strength can reduce forced vital capacity (FVC) and induce restrictive lung dysfunction (5). Additionally, impaired cough reflex and reduced airway clearance capacity elevate the risk of atelectasis, potentially culminating in respiratory failure (6). Currently, there is no effective curative treatment for this disease, and management primarily relies on supportive and symptomatic measures to control disease progression and alleviate symptoms. Although some studies suggest that immunosuppressive therapy or corticosteroids may offer certain benefits, the effects are often transient and lack robust clinical evidence (7).

While the characteristics and diagnostic criteria for IBM are well established (8), specific anesthetic guidance for these patients remains limited. This challenge is even more pronounced in the rare scenario of pregnant patients. Although IBM does not affect the uterus, IBM patients face heightened respiratory danger during pregnancy and labor, attributable to diaphragmatic elevation and fatigue of the voluntary muscles, which may necessitate cesarean delivery in some cases (8). This case report demonstrates the safe use of general anesthesia for cesarean section in a parturient with IBM, offering valuable guidance for the anesthetic and perioperative management of this rare clinical scenario.

## Case presentation

**Figure 1:**
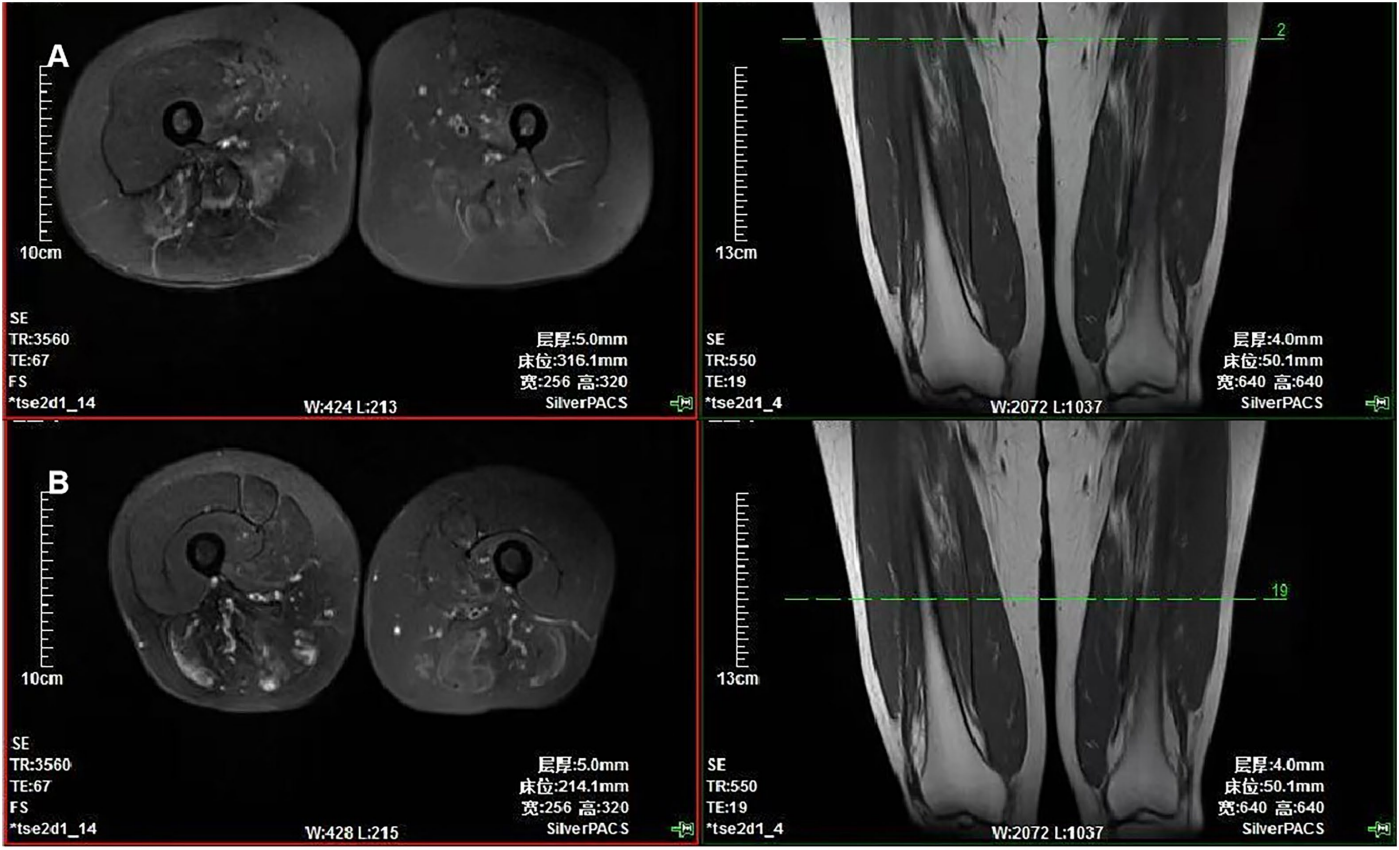
MRI of the patient’s bilateral femoral muscles: **(A)** muscular atrophy; **(B)** muscular edema.

A 43-year-old parturient (G2P1) with a height of 160 cm and a weight of 80 kg presented at 39 weeks’ gestation. The patient had been diagnosed with IBM 7 years prior to this pregnancy and had developed gestational diabetes mellitus during the current pregnancy (Supplementary Material 1). No systematic treatment was administered during this period. She had become wheelchair-dependent 4 years ago due to progressive lower extremity muscle weakness and required assistance with activities of daily living. On admission, the patient’s muscle strength of both upper limbs was Grade IV, the proximal muscle strength of both lower limbs was Grade II, along with marked quadriceps muscle atrophy (Figure 1). The patient had mild dysphagia due to IBM, with no significant respiratory muscle involvement. Pulmonary function test results (Table 1), electrocardiogram, blood counts and blood chemistries analyses were unremarkable. The patient’s serum creatine kinase (CK) level was 377.4 U/L (reference range <200 U/L). Ultrasonographic fetal biometry revealed an abdominal circumference (AC) of 37.4 cm and an estimated fetal weight (EFW) of 4,223 ± 417 g. These values met the diagnostic criteria for fetal macrosomia, prompting the decision to perform cesarean delivery.

**Table 1:** Pulmonary function test results of the patient.

Parameter	Predicted value	Measured value	Measured/predicted (%)
VC MAX (L)	3.25	3.42	105.6
VT (L)	0.57	0.90	156.7
FVC (L)	3.17	3.42	107.9
FEV 1 (L)	2.72	2.98	109.4
FEV 1/FVC (%)		87.16	
MMEF 75/25 (L/s)	3.48	3.74	107.3
MVV (L/min)	102.24	91.05	89.1
RV (L)	1.62	2.15	132.7
TLC (L)	4.90	5.63	114.8
RV/TLC (%)	33.58	38.21	113.8

The key anesthetic considerations are summarized as aspiration risk, susceptibility to malignant hyperthermia (MH), exaggerated sensitivity to neuromuscular blocking agents (NMBAs), and predisposition to postoperative pulmonary complications.

**Figure 2:**
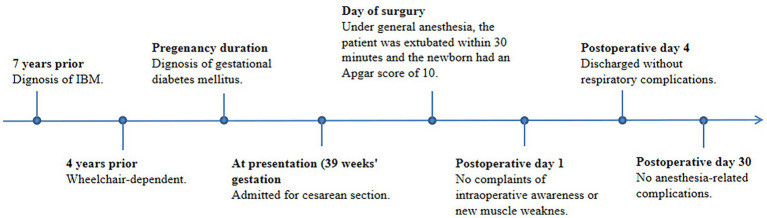
Timeline of the patient process. IBM, inclusion body myositis.

The following monitors were attached to the patient: electrocardiogram, pulse oximeter, noninvasive blood pressure, peripheral nerve stimulator, and core body thermometer. The preoperative aspiration prophylaxis protocol consisted of the following: (1) an 8-h fasting period; (2) administration of a non-particulate antacid; and (3) maintenance of the patient in a head-elevated position throughout the procedure. Specific preventive measures against MH carried out on the day of surgery were as follows: (1) physical removal of vaporizers and any residual volatile anesthetics from the anesthesia delivery system; (2) utilization of a fresh, non-rebreathing circuit; (3) placement of charcoal filters on the inspiratory and expiratory valves, followed by flushing the system with 100% oxygen; (4) avoidance of all MH-triggering agents (e.g., inhalational anesthetics and succinylcholine); and (5) confirmation of dantrolene availability for emergent use in the event of MH. The patient was preoxygenated, and cricoid pressure was applied prior to rapid sequence induction with remifentanil (1 μg/kg), propofol (2 mg/kg), and a reduced intubating dose of rocuronium (0.4 mg/kg). Anesthesia was maintained with propofol and remifentanil infusions at 4–10 mg/kg/h and 0.05–0.2 μg/kg/min, respectively. Additional doses of rocuronium at 0.1 mg/kg were given based on neuromuscular blockade monitoring. The fetus was delivered 3 min after the initiation of surgery. The newborn was assigned a 10/10 Apgar score at 1 and 5 min after delivery, with a birth weight of 4,390 g. Intravenous acetaminophen was administered for postoperative analgesia. At the end of surgery, neuromuscular blockade was reversed with sugammadex (2 mg/kg) and a 100% train-of-fourratio was achieved. The patient established regular breathing and an adequate spontaneous minute ventilation, and emerged to an awake state with eye-opening and ability to follow commands, thereby meeting extubation criteria (9). The patient was extubated within 30 min and transferred to her room, where her condition remained stable (Supplementary Video 1). The patient had no complaints of intraoperative awareness or new muscle weakness on the first postoperative day. She was discharged on the fourth day after the operation without respiratory complications. No anesthesia complications occurred within 30 days after surgery (Figure 2).

## Discussion

Published studies focusing on surgical interventions in patients with IBM have tended to emphasize risks and complications associated with the disease itself or anesthetic management, rather than those directly deriving from the surgical procedure. Previous studies have explored the use of neuraxial anesthesia for surgical procedures in patients with neuromuscular disorders, including IBM (10). However, when respiratory muscles are affected, these patients still require ventilator assistance during surgery (10, 11). Recent studies have reported that general anesthesia can be safely administered in IBM patients. In an observational study of 18 IBM patients undergoing general anesthesia, 13 patients were extubated immediately after surgery without exacerbation of muscle weakness, while 5 patients required delayed extubation for planned postoperative respiratory support (12). A case report documented an IBM patient who underwent esophagoscopy and percutaneous endoscopic gastrostomy under general anesthesia. This patient was successfully extubated 1 hour postoperatively without any respiratory complications (13). Similarly, a reported IBM patient underwent general anesthesia for coronary artery bypass grafting, was administered sugammadex postoperatively, and was extubated within 1 hour with no new-onset muscle weakness reported (14). However, as clinical symptoms progress, general anesthesia carries increased risks. A case of an IBM patient with type II chronic respiratory failure underwent thoracoscopic surgery under general anesthesia. Despite reversal of neuromuscular blockade, the patient could not be extubated immediately after surgery (15). Thus, the degree of muscular involvement in IBM patients determines the risk of general anesthesia-related complications.

In our case, general anesthesia was selected over neuraxial anesthesia for the patient with IBM for several reasons. The patient’s progressive lower extremity muscle weakness, along with pregnancy-related abdominal enlargement, made it difficult to maintain the lateral decubitus position required for neuraxial puncture, which could lead to puncture failure. Furthermore, neuraxial anesthesia carries a potential risk of nerve injury complications, such as cauda equina syndrome and needle-induced mechanical nerve injury. Moreover, neuraxial anesthesia itself might exacerbate the patient’s underlying neuromuscular dysfunction, making it difficult to differentiate postoperative conditions from the patient’s pre-existing myopathy. In summary, given that the patient had no respiratory muscle involvement, general anesthesia could be performed more safely.

The CK levels of this patient were elevated, indicating heightened susceptibility to MH; thus inhalational anesthetics and succinylcholine were avoided in this case (16). During the operation, we ensured the ready availability of MH-rescue equipment, supplies, and medications, especially dantrolene, to prepare for any potential MH crisis. Rocuronium was selected as the muscle relaxant to eliminate the risk of triggering MH. In our case, propofol and remifentanil were administered, as they have a rapid onset of action, a short duration of effect, and allow for quick recovery. Although both agents can cross the placenta, they are rapidly metabolized. Recent studies have reported that remifentanil appears to be safe when used as an induction agent for cesarean section under general anesthesia, with no significant effect on Apgar scores, and that it attenuates the maternal circulatory response to intubation and surgery (17). A reasonable body of evidence supports the choice of propofol as the induction agent for general anesthesia in cesarean section in healthy, non-compromised patients, because propofol is associated with less intraoperative awareness than thiopental (18).

In both the current and prior cases, reduced doses of NMBAs were administered, or the agents were avoided entirely, in IBM patients owing to their theoretically enhanced sensitivity to these drugs. However, beyond anecdotal clinical experience, there are no data to definitively establish the pharmacokinetics, pharmacodynamics, and relative safety of NMBAs in this setting (19, 20). Administering a decreased dose for shorter cases may be prudent due to decreased pulmonary function and potential for prolonged ventilatory support in certain patients with IBM. Consistent with this principle, we adopted a reduced-dose NMBA strategy for the present short-duration cesarean section. Real-time neuromuscular function monitoring and sugammadex were applied in this case and our patient achieved uneventful extubation within 30 min.

A further clinical concern pertaining to IBM patients undergoing anesthesia is the potential for aspiration. Given that our patient had mild dysphagia, we instituted a full set of aspiration prophylaxis measures, namely the cricoid pressure application, an 8-h preoperative fast, administration of a non-particulate antacid, and maintenance of the patient in a head-elevated position throughout the procedure. As a direct result of these interventions, no aspiration events occurred throughout the perioperative period.

## Conclusion

This case demonstrates that, with precautionary preparations targeting potential complications—including aspiration risk, MH, exaggerated sensitivity to NMBAs, and postoperative pulmonary complications—general anesthesia may be a safe and feasible option for cesarean delivery in IBM patients. Unfortunately, this case report lacks long-term follow-up. Without assessment at longer postoperative time points (e.g., 6 months), we are unable to determine whether the anesthetic management strategy had any delayed effect on the progression of disease-related muscle weakness or respiratory function, nor to fully track the long-term outcomes of both the mother and the newborn. Further large-scale, prospective studies are therefore required to validate these preliminary findings and establish standardized anesthetic management for this patient cohort.

## Data Availability

The original contributions presented in the study are included in the article/Supplementary material, further inquiries can be directed to the corresponding authors.

## Supplementary material

SUPPLEMENTARY MATERIAL 1 Pathological report of muscle biopsy.

SUPPLEMENTARY VIDEO 1 Postoperative video.
